# Current News

**Published:** 1895-12

**Authors:** 


### Current News.

### REPORT OF TREASURER OF WORLD'S COLUMBIAN DENTAL CONGRESS IN FULL FROM MARCH 23, 1891, TO JULY 23, 1895.

RECEIPTS.

| | |
|---|---|
| American Dental Association | $1,000 00 |
| Illinois State Dental Society | 321 00 |
| Iowa State Dental Society | 100 00 |
| Southern Dental Association | 200 00 |
| Washington City Dental Society | 50 00 |
| Alabama | 130 00 |
| Arizona | 20 00 |
| Arkansas | 20 00 |
| Austria | 10 00 |
| California | 548 00 |
| Canada | 20 00 |
| Chili | 10 00 |
| China | 30 00 |
| Colorado | 40 00 |
| Connecticut | 220 00 |
| Cuba | 10 00 |
| Delaware | 50 00 |
| District of Columbia | 190 00 |
| England | 50 00 |
| Florida | 20 00 |
| France | 100 00 |
| Georgia | 125 00 |
| Germany | 10 00 |
| Hawaii Islands | 40 00 |
| Illinois | 3,802 00 |
| Indiana | 355 00 |
| Iowa | 460 00 |

| | |
|---|---|
| Italy | 10 00 |
| Kansas | 130 00 |
| Kentucky | 120 00 |
| Louisiana | 60 00 |
| Maine | 190 00 |
| Maryland | 250 00 |
| Massachusetts | 395 35 |
| Mexico | 20 00 |
| Michigan | 245 00 |
| Minnesota | 405 00 |
| Miscellaneous | 11 70 |
| Mississippi | 40 00 |
| Missouri | 715 00 |
| Montana | 10 00 |
| Nebraska | 180 00 |
| New Hampshire | 10 00 |
| New Jersey | 477 50 |
| New South Wales | 30 00 |
| New York | 1,515 30 |
| North Carolina | 210 25 |
| North Dakota | 40 00 |
| Ohio | 620 00 |
| Oregon | 20 00 |
| Paraguay | 20 00 |
| Pennsylvania | 840 30 |
| Rhode Island | 25 00 |
| Russia | 30 00 |
| Scotland | 20 15 |
| South America | 10 00 |
| South Carolina | 130 00 |
| South Dakota | 10 00 |
| Spain | 20 00 |
| Switzerland | 20 00 |
| Tennessee | 290 00 |
| Texas | 170 00 |
| Vermont | 120 00 |
| Virginia | 100 00 |
| Washington | 80 00 |
| West Virginia | 10 00 |
| Wisconsin | 150 00 |
| American College of Dental Surgery | 10 00 |
| Baltimore College | 10 00 |
| Chicago College | 10 00 |
| Columbia University | 10 00 |
| Louisville Dental College | 20 00 |
| New York Dental College | 10 00 |
| Ohio Dental College | 10 00 |
| Philadelphia College | 10 00 |
| University of Buffalo | 10 00 |

| | |
|---|---|
| University of California | 10 00 |
| University of Minnesota | 10 00 |
| University of Western Reserve | 10 00 |
| Wilmington Dental Manufacturing Company | 40 00 |
| | $15,851 55 |
| Amount received from Columbia National Bank | 286 06 |
| Donations of Executive Committee | 3,600 00 |
| | $19,737 61 |

DISBURSEMENTS.

| | |
|---|---|
| General Finance Committee, L. D. Shepard | $926 25 |
| Executive Committee, Secretary's Expenses | 3,018 84 |
| Treasurer's Expenses | 583 59 |
| Treasurer's Bond | 100 00 |
| Secretary's General Expenses | 1,299 48 |
| State Conference Committee, J. Taft | 218 87 |
| Registration Committee, Fred. A. Levy (per G. C. Brown) | 34 00 |
| Invitation Committee, W. C. Barrett | 16 95 |
| Clinics Committee, C. F. W. Bödecker and S. H. Guilford | 80 35 |
| Biology Committee, R. R. Andrews | 18 15 |
| Nomenclature Committee, G. V. Black | 31 00 |
| History Committee, J. Taft | 1,523 57 |
| Membership Committee, E. Noyes | 13 50 |
| Publication of Transactions Committee, A. W. Harlan | 4,529 95 |
| Donations by Executive Committee, Travelling Expenses | 3,600 00 |
| Woman's Department | 307 00 |
| Dental Manufacturing Company, S. S. White | 121 80 |
| Medals | 720 00 |
| Buttons and Badges | 257 63 |
| Club House | 1,530 C0 |
| Banquet, Deficit | 274 60 |
| Refunded Memberships | 50 00 |
| Exchange | 6 36 |
| | $19,261 89 |
| Paid by Columbia National Bank | 286 06 |
| Unpaid by Columbia National Bank | 127 09 |
| | $19,675 04 |
| Balance, July 23, 1895, in Merchants' Loan and Trust Company Bank | 62 57 |
| | $19,737 61 |

(Signed) JOHN S. MARSHALL,
*Treasurer.*

### MASSACHUSETTS DENTAL SOCIETY.

The following officers have been elected for the ensuing year: President, Geo. A. Maxfield, D.D.S., Holyoke; First Vice-President, Waldo E. Boardman, D.M.D., Boston; Second Vice-President, Harry S. Draper, D.D.S., Boston; Secretary, Edgar O. Kinsman, D.D.S., Cambridge; Treasurer, Edward Page, M.D., D.M.D., Charlestown District; Librarian, Thomas W. Clements, D.D.S., Brookline; Editor, Joseph T. Paul, D.M.D., Boston.

The State and Society have been divided into districts, with officers governing the same, as follows: North and South Metropolitan, North and South Eastern, Central, Valley and District Dental Societies, Western.

Edgar O. Kinsman, M.D.S.,
*Secretary.*

---

### GOLDEN ANNIVERSARY CELEBRATION.

Philadelphia, November, 1895.

Dear Doctor,—Fifty years ago the first effort was made to organize the dentists in Philadelphia by forming a society having for its object the advancement of dental education.

You are cordially invited to attend a meeting and banquet to celebiate the golden anniversary of that event, to be held at the Continental Hotel, Ninth and Chestnut Streets, on Monday, December 16, 1895, at 7.00 p.m. Banquet at 7.30 p.m.

Kindly inform us by December 10 if we may expect the pleasure of your company.

Seats for the banquet will be $4.00 per plate, which amount please send to Dr. J. D. Thomas, 912 Walnut Street, Philadelphia, by December 13, in order that a place may be reserved for you.

COMMITTEE.

*Pennsylvania Association of Dental Surgeons.*—Dr. Wm. H. Trueman, Dr. T. F. Chupein, Dr. Howard E. Roberts.

*Odontological Society.*—Dr. L. Ashley Faught, Dr. Alonzo Boice, Dr. I. N. Broomell.

*Academy of Stomatology.*—Dr. James Truman, Dr. R. Huey, Dr. Henry Register.

*Special Committee on Invitations.*—Dr. L. Ashley Faught, Dr. Howard E. Roberts, Dr. J. D. Thomas, *Chairman*, 912 Walnut Street.

